# Adaptiveness for Online Learning: Conceptualising ‘Online Learning Dexterity’ from Higher Education Students’ Experiences

**DOI:** 10.1007/s40841-023-00287-2

**Published:** 2023-05-13

**Authors:** Joyce Hwee Ling Koh, Ben Kei Daniel, Angela C. Greenman

**Affiliations:** 1 Higher Education Development Centre, University of Otago, 65-75 Union Place West, PO Box 56, Dunedin, 9054 New Zealand; 2 Department of Cellular and Molecular Medicine, University of Arizona, Tucson, AZ 85724 USA

**Keywords:** Online learning dexterity, Online learning competency, E-learning competency, COVID-19, Self-regulation, Student experiences

## Abstract

Online learning dexterity, or the ability to effortlessly adapt to online learning situations, has become critical since the COVID-19 pandemic, but its processes are not well-understood. Using grounded theory, this study develops a paradigm model of online learning dexterity from semi-structured interviews with 32 undergraduate and postgraduate students from a university in New Zealand. Through students’ online learning experiences during the pandemic from 2020 to 2021, online learning dexterity is found to be how students make online learning ‘just as good’ as face-to-face learning by creating and adjusting five learning manoeuvres according to developing online learning circumstances. Undergraduates and postgraduates re-use familiar study strategies as *deep learning manoeuvres,* but undergraduates restrict *support-seeking manoeuvres* to lecturers. Technical problems with online systems and poor course organisation by lecturers affected learning productivity, resulting in the need for more *time optimisation manoeuvres*. Social support helped students activate *persistence manoeuvres* to sustain online class attendance. However, undergraduates had more problems sustaining interest and engagement during class as they were not as proficient with using *learning presence manoeuvres* as postgraduates enrolled in distance learning programmes. The theoretical and practical significance of online learning dexterity for post-pandemic higher education is discussed.

## Introduction

Dexterity refers to the quality of hand skills, especially regarding flexibility and agility (Merriam-Webster, n.d.). This study considers what dexterity could mean when students learn online. This could be especially pertinent after higher education institutions had to rapidly shift teaching and learning online during the COVID-19 pandemic (the pandemic). Numerous reports indicate students having challenges with this new way of learning (e.g. Biwer et al., 2021; Marshalsey & Sclater, 2020). While some of their struggles could arise from personal or infrastructural factors, students may also lack what we term ‘online learning dexterity’ or the ability to adapt to different situations during online learning (Koh & Daniel, 2022).

Adaptiveness is a form of expertise (Hatano & Inagaki, 1986). Adapting to changing learning environments may be difficult because even students enrolled in distance learning programmes become competent in online learning only after progressively adjusting their learning strategies (Lee et al., 2019). Students’ need for online learning dexterity may not diminish after the pandemic because higher education institutions are predicted to continue expanding learning options via entirely online, blended, or hybrid learning arrangements (EDUCAUSE, 2022). Yet, online learning dexterity has not been explicated in current literature. Few studies examine online students’ actual learning processes, while those available focus on surveying students' perceptions of self-regulation and online learning success (e.g. Broadbent & Poon, 2015; Neroni et al., 2019).

In this study, we attempt to demarcate the meaning of online learning dexterity by using grounded theory to construct a situated view of students’ online learning experiences (Corbin & Strauss, 1990). Through semi-structured interviews with 32 students from a university in New Zealand, we first conceptualise a framework of online learning dexterity by understanding students’ perspectives, how they learnt online, their adjustments, and their study experiences during the pandemic from 2020 to 2021. We then discuss the theoretical and practical implications for higher education.

## Literature Review

In higher education, online learning refers to courses delivered synchronously or asynchronously through the Internet (Singh & Thurman, 2019). Researchers have been particularly interested in online students’ self-regulation because teachers and students tend to be physically separated during online learning. Zimmerman (2002) defined self-regulation as students’ ability to sustain learning goals through planning, executing, evaluating, and improving their performance of learning tasks independently. Zimmerman also suggests that students self-regulate with cognitive, metacognitive, and resource management strategies. Some studies have appropriated Zimmerman’s three self-regulation strategies as online learning strategies (e.g. Hu & Gramling, 2009; Neroni et al., 2019), premising that online students use cognitive strategies to review and organise content information, metacognitive strategies to assess task performance and modify task strategies, and resource management strategies to manage time, effort, and learning support (see Mayer, 1988; McCombs, 2017).

Self-regulation may not fully capture the idea of online learning dexterity because it concerns how one sets goals and manages the strategies for completing learning tasks (Pilling-Cormick & Garrison, 2007). The few available qualitative studies of mature students’ experiences in distance learning programmes show that online learning goes beyond how they self-regulate their immediate learning tasks. These students use self-regulation strategies for metacognition and resource management at the beginning stages of online learning to establish study routines. As their online learning progressed, these students continually adjusted how they used institutional technology systems, projected their online presence in different learning platforms, managed feelings of isolation, and sustained learning motivation (Lee et al., 2019; Symeonides & Childs, 2015). These findings show that online learning strategies are not static but need to be continually refined. Therefore, we see online learning dexterity as involving *how* students manoeuvre and adjust to the different circumstances and situations that they may encounter during online learning with deftness. This aspect of students’ online learning experiences is not well-understood because of a general dearth of qualitative studies. The plethora of statistical findings from survey studies, primarily based on self-regulation strategies, are also inconsistent. For example, some studies found that resource management and cognitive strategies have significant effects on online learning performance (e.g. Hu & Gramling, 2009; Neroni et al., 2019), but a systematic review by Broadbent and Poon (2015) found significant effects for metacognitive strategies while only resource management strategies associated with time and effort, and cognitive strategies associated with critical thinking were significant. What online learning dexterity could mean in students’ actual online learning practices has not yet been conceptualised in current literature.

Post-pandemic, students may not need to adjust to studying and living in lockdown situations (e.g. Dietrich et al., 2020). Improved access to on-campus infrastructure could also mean fewer challenges for students to access computing devices and bandwidth for online studies (e.g. Yundayani et al., 2021). However, the role of online learning in higher education may have been subtly changed by emergency remote teaching during the pandemic. Institutions that could convert on-campus courses online—even those involving clinical, field-based, and studio-based training (Chan et al., 2020; Dodson & Blinn, 2021; Gerhart et al., 2021), may be better able to reach untapped student markets. Lecturers may also be keen to continue with online activities that proved successful during emergency remote teaching (EDUCAUSE, 2022). The ebbing pandemic may not mean a simple reversion to classroom learning. There is a possibility that online learning could become more entrenched or even a permanent feature of higher education.

Literature published before the pandemic more often captured the experiences of mature students enrolled in asynchronous distance learning courses (e.g. Symeonides & Childs, 2015). This may not adequately address the complexities of current online learning contexts. Institutions shifted online rapidly during the pandemic by replacing on-site class hours directly with video conferencing (Koh & Daniel, 2022), but this exacerbated students’ screen fatigue, challenging them to find ways of maintaining concentration (e.g. Yeung & Yau, 2021). Students also had to manage different modes of online learning. Undergraduates found it particularly challenging to consistently learn asynchronously with online resources (e.g. Marshalsey & Sclater, 2020). However, during synchronous video conferencing, students turned off their webcams and microphones, making it difficult for teachers to encourage interaction and engagement (Castelli & Sarvary, 2021). Shifting online during the pandemic has not compromised students’ learning outcomes, but a systematic review found that learning experiences were not optimal for all (Koh & Daniel, 2022). It appears that students still need better online learning competencies. However, such competencies are contextualised rather than standardised (Alvarez et al., 2009) and highly influenced by factors such as institutional technology environments and teachers’ design of online lessons (Lee et al., 2019). How students adapt their learning strategies according to the online learning situations and circumstances they encounter are critical learning competencies that have yet been clearly understood. A clearer demarcation of online learning dexterity can address this research gap.

## Research Goals

We articulate two research goals from the above review as follows:

- Understanding the conception of online learning dexterity from students’ perspectives of their online learning experiences and strategies
- Describing the different ways that students demonstrate online learning dexterity, if any

## Methods

Grounded theory was chosen as the research methodology because it is based on pragmatism, which allows for the social phenomenon of online learning dexterity to be examined through higher education students’ experiences and perspectives (Creswell, 1998). Since online learning dexterity has not been clearly defined in current literature, the analysis methods of grounded theory support us to theorise it through articulating its key concepts and their inter-relationships (Charmaz, 2006). An interpretive perspective of grounded theory attributed to Corbin and Strauss (1990) is adopted as we recognise that the arising theory is a product of our interpretation and framed within participants’ experiences of online learning in their institutional contexts. This grounded theory perspective guides theorisation through the construction of a paradigm model to capture the interplay among strategies, contextual variables, and outcomes (Sebastian, 2019). It aligns with our research goal of capturing an in situ view of students’ pandemic online learning experiences.

### Study Context and Data Collection

Data were collected from undergraduate and postgraduate students studying at a public university in New Zealand in 2020 and 2021. We recruited study participants from one university to better control how contextual factors such as learning management systems and course policies may influence students’ online learning practices (Koh & Kan, 2020). Since March 2020, the university has been adjusting class arrangements in response to the Alert levels announced by the New Zealand government (Unite against COVID-19, 2022). On-campus students learnt entirely online during Alert Level 4 lockdowns and progressively shifted back to campus-based learning from September 2020. The changing Alert levels did not affect course arrangements in distance learning programmes, but since distance learning students were mainly working adults, the pandemic may have some impact on their work and study arrangements.

After approval by the university's ethics board, recruitment emails for voluntary participation in a one-hour semi-structured interview were sent to students via their faculty offices. The study information was explained to each volunteer, who signed informed consent forms before participating in the interviews. The first and third authors conducted the interviews between April to July 2021. Depending on the participants’ preferences and the general social distancing guidelines of the university, both online and face-to-face interviews were conducted. The interview questions were designed to facilitate constructive conversations in line with the grounded theory approach (Corbin & Strauss, 1990). Participants were invited to share their 2020 and 2021 pandemic learning experiences chronologically and were asked to describe their learning strategies, how they created successes and coped with online learning challenges during different pandemic stages. We also explored with participants their conceptions of online learning dexterity. All interviews were audio-recorded and transcribed to text.

### Sampling and Data Analysis

Given the paucity of online learning dexterity studies in the extant literature, we began the study with maximum variation sampling (Morse & Clark, 2019), seeking to understand the phenomenon through different disciplinary contexts. There are few prescribed guidelines regarding an optimal sample size for grounded theory studies because sampling is taken to be an emergent process whereby study participants are purposively and continually added until there is theoretical saturation (Charmaz, 2006). Therefore, students were recruited for voluntary participation through the four key divisions of the university, i.e., Commerce, Health Sciences, Sciences, and Humanities. As per the interpretive perspective of grounded theory, we began data analysis after each interview (Corbin & Strauss, 1990). At the same time, we continued to recruit students, particularly to reach divisions with fewer volunteers and students of different profiles, such as undergraduates, first-year students, mature students, distance learning students, postgraduates, indigenous students, and international students, for maximum variation.

Data were transcribed and cleaned after each interview, following which open coding began with NVIVO. Each interview transcript was coded line by line and interpreted as concepts (termed as references in NVIVO) that were then assigned into categories (termed as codes in NVIVO) by constant comparison (Charmaz, 2006; Corbin & Strauss, 1990). Several strategies were used to ensure reliability and trustworthiness during open coding (Creswell, 1998). Firstly, the researchers had weekly peer review meetings, and memos were used to capture and document how the team reviewed ideas and problems associated with coding. Secondly, a systematic literature review was carried out for empirical insights into ways of inter-relating emerging categories (Koh & Daniel, 2022; Sebastian, 2019). Thirdly, an audit trail was maintained whereby the memos were updated to document how concepts and categories were re-labelled, merged, or removed throughout these processes. Through this open coding process, we eventually recorded 60 codes from 2606 references in NVIVO.

The coding paradigm of Corbin and Strauss (1990) suggests that theories can be developed by examining the contexts of a phenomenon, its causal conditions, its associated strategies and their consequences, and possible intervening conditions. Using this paradigm as a reference, the codes derived from open coding were organised through axial coding to articulate the learning strategies undergirding online learning dexterity, the consequences of these strategies, and their possible causal and intervening conditions. We progressively used axial coding to develop plausible relationships among categories, as these developed during open coding. Concurrently, we used theoretical sampling to improve the theoretical saturation of categories by purposively recruiting students from centres providing academic support, distance learning support, and support for indigenous students, examining their experiences against the coding paradigms we developed (Corbin & Strauss, 1990). Through this process, we eventually interviewed 32 students (See profile in next section).

To ensure reliability and trustworthiness during axial coding, memoing (Charmaz, 2006) took the form of reports consolidating the major themes observed from axial coding. These reports were summarised by divisions of study, i.e., Commerce, Health Sciences, Sciences, and Humanities and sent to the study participants for review as a form of member checking (Creswell, 1998). Feedback from programme administrators of different departments of study and support centres was also gathered to refine the themes observed.

During selective coding, the divisional reports were examined for common themes and visualisations of the paradigm model were created and reviewed among the authors. The first author wrote the storyline accompanying the visualisation, and a critical peer review was carried out among the researchers to derive the theorisation of online learning dexterity outlined in the findings.

### Participant Profile

Table 1 outlines the participant profile. About 60% of the participants were undergraduates enrolled in on-campus courses. The majority of the distance learning student were also postgraduates.

**Table 1:** Profile of study participants

	N	%
*Division of study*		
Humanities	11	34
Sciences	4	13
Health Sciences	8	25
Commerce	9	28
Total	32	100
*Gender*		
Male	17	53
Female	15	47
Total	32	100
*Level of study*		
Undergraduate	20	63
Postgraduate	12	37
Total	32	100
*Course enrolment*		
Distance learning	12	37
Undergraduate	1	
Postgraduate	11	
On-campus	20	63
Undergraduate	18	
Postgraduate	2	
Total	32	100

## Findings

### Conceptions of Online Dexterity

**Fig. 1:**
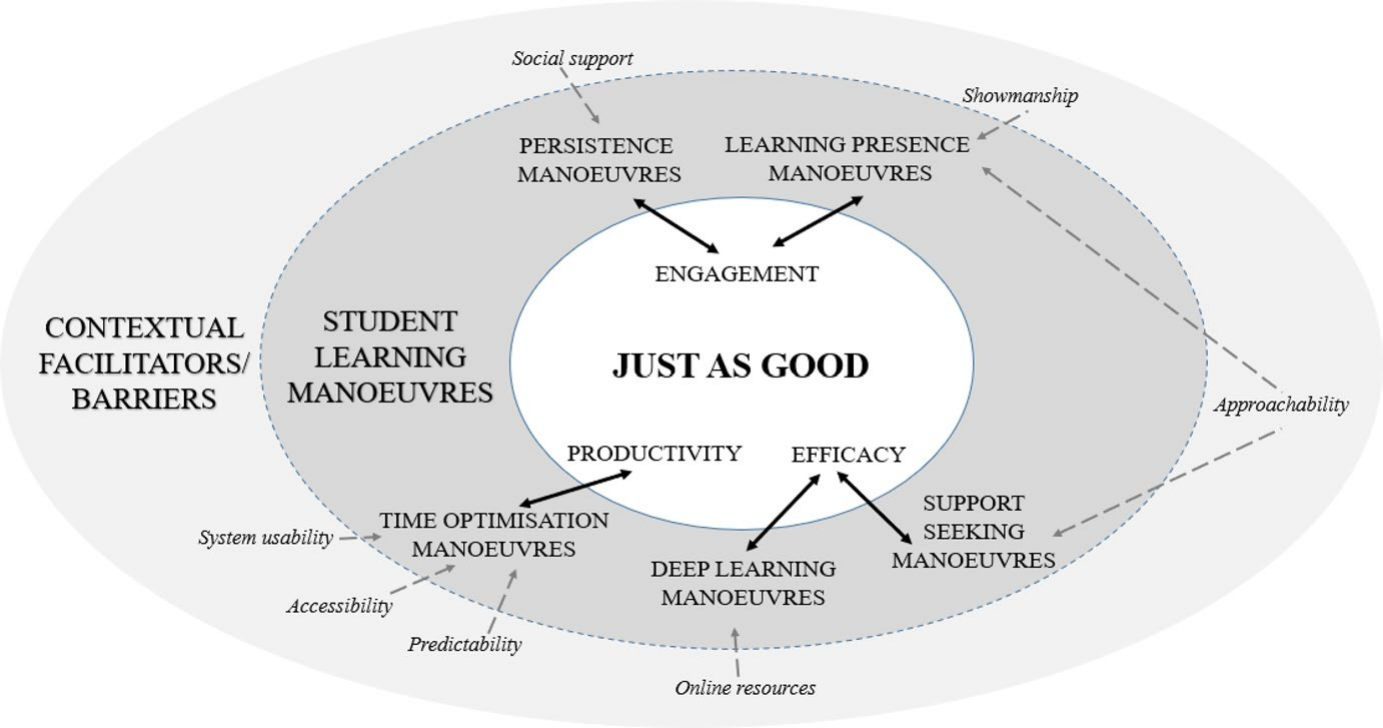
Paradigm model of online learning dexterity

From students’ pandemic learning experiences, online learning dexterity encapsulates how they used learning manoeuvres to make online learning *just as good* as face-to-face learning where "… I did not feel there was a huge loss in having it all be online.” Students interpreted the core category (Corbin & Strauss, 1990) of *just as good* in three ways. Firstly, it meant productivity whereby students could “quickly get [a] hold of everything I wanted [content resources] … within seconds.” Secondly, it was about having efficacy where “… you've actually learnt something.” Thirdly, it was about having engagement rather than “just sitting here with my headphones listening to that same voice while the slides are skipping across my screen.” Students displayed online learning dexterity differently as they tried to sustain these three dimensions of *just as good*. We observe this process to often be emergent and episodic. Students appeared to be groping and manoeuvring according to the situation while responding to contextual facilitators and barriers (See paradigm model in Fig. 1). Therefore, we used the term ‘learning manoeuvres’ to categorise these situational tactics underpinning students’ online learning dexterity.

### Online Learning Dexterity for Productivity

The online learning process should be just as productive for the students because it should not take them more time to complete classroom study tasks online. However, students had to adapt their online study processes with new ‘time optimisation manoeuvres’. As it is vital to know “where to go to find your lectures, how to upload stuff,” students invested time to become proficient with institutional systems. They also found ways to use lecture recordings*.* Some undergraduate students listened to podcasts of lectures while “in a bus or walking”, while others used lecture recordings to buy more work time when assignments were due. An on-campus student shared, “What is the incentive to go [to a live lecture]? … [if] I am rushing to finish the assignment … it is gonna punish my grades overall.”

Several contextual factors helped or hindered students’ time optimisation manoeuvres. ‘System usability’ was “absolutely vital, like a skeleton.” Students felt that “… the tool's [learning management system’s] usability for both teachers and students can make or break the experience.” For example, when accessing lecture videos, “I want to skip seven minutes ahead, and suddenly there's an issue.” Consequently, this student wasted more time replaying the whole video.

How lecturers designed course sites with ‘accessibility’ was also critical. When lecturers “laid out content [in a] logical sequence,” it facilitated productivity because “everything was just one click to get there.” When “every paper [course] was different on Blackboard™ … they have their own words [to label resources such as] lecture slides,” students had to spend extra time deciphering course structures.

‘Predictability’*,* or whether an online course had stable “policy and procedure”, influenced how students used time optimisation manoeuvres. For example, when classes were entirely online during the lockdown, how lecturers released lecture recordings influenced students’ study schedules. When there was irregular uploading where “… one week, it is 11 [o’clock] … and the next week it is five o’clock” meant lower productivity because students spent extra effort adjusting study schedules with time optimisation manoeuvres. Students found this inefficient and devalued online learning because “If there wasn’t consistency from your lecturer, … it would have been [easier] had you just been at [the] uni[versity].” Assessment design contributed to predictability and influenced students’ time optimisation manoeuvres. For an on-site exam, students would do “two hours [of] writing [and] this would be over.” Converting this to an essay assignment stretched assessment across the semester, which also meant more effort into using time optimisation manoeuvres to plan and balance work schedules.

### Online Learning Dexterity for Efficacy

Students recognised that they did not study differently online as they tended to re-use the study strategies that were already working for them. Therefore, students' ‘deep learning manoeuvres’ for achieving “more comprehensive” content understanding comprised their tried-and-tested strategies for note-taking and revision. However, there were qualitative differences between the deep learning manoeuvres used by undergraduates and postgraduates. Postgraduates did additional research after class and synthesised what they learnt by “re-arrang[ing] the content” to form personal conceptual maps. Before the pandemic, those already enrolled in distance learning realised that they needed to fine-tune their deep learning manoeuvres to sustain efficacy in online learning environments. For example, a postgraduate noticed that during live Zoom™ sessions. “… [I struggled with] writing notes whilst listening in an online environment—it was [a] distraction.” Therefore, there was a need to consider, “Do you take notes? Do you just listen? … Do you interact?” and whether “just focusing on the lecture and paying attention to the material” helped them to realise better efficacy through their deep learning manoeuvres. In comparison, undergraduates focused on exam revision and primarily used lecture recordings, fast forwarding to “…the important slides and listen to it.”

The availability of ‘online resources’ could serve as a contextual facilitator of students’ deep learning manoeuvres. Postgraduates actively expanded their deep learning manoeuvres by analysing “the [online] resources available to you and how you can use them.” They also posted questions and searched for answers on the class online discussion board. Undergraduates admitted that they focused on lecture recordings and did not keep abreast of the other resources that lecturers made available. When they did, they found interactive resources such as “a small video of the [laboratory] procedure, and you could just scroll through it and do the questions and make the analysis you needed” useful.

Students also supplemented deep learning manoeuvres with ‘support seeking manoeuvres’ to sustain efficacy*.* Undergraduates believed they could better understand “when I actually talk to the teacher.” While they were generally “very shy to ask for help” and hesitant even if they were “struggling,” they took the initiative and were “emailing my lecturer or tutor to ask for more information” while studying online during the lockdown. When lecturers provided them with opportunities for peer support through Zoom™ breakout room discussions, undergraduates found that “… in the absence of having [the] lecturer or somebody to guide the discussion… we just didn't really know what to do.” In comparison, postgraduates took the initiative to expand support-seeking manoeuvres through learning online with peers. They valued opportunities to “get your head around a topic with four or five other people in your cohort” in breakout room discussions and described this as the “best part” of their online learning experiences.

Having ‘approachability’ or “human touch points” in the learning environment could encourage students’ support-seeking. Postgraduates found ways to create approachability within their learning groups. For example, after online sessions ended, some groups “stayed online for an hour and a half, and just chatted … these are friends.” Conversely, undergraduates relied more on lecturers to create approachability by providing them with different ways to “access the lecturer to talk through a few things.”

### Online Learning Dexterity for Engagement

How students used ‘persistence manoeuvres’ and ‘learning presence manoeuvres’ influenced their online learning engagement. Persistence manoeuvres were actions that helped students to sustain the right frame of mind for enthusiastic online participation. Distance learning students’ online learning experiences before the pandemic made them realise the importance of having “discipline” and “resilience”. However, keeping focused on their study goals was a persistence manoeuvre that helped them to continue studying amidst pandemic stressors on their job security and family situations. Undergraduates mainly enrolled in on-campus programmes realised the need to be more purposive about persistence manoeuvres during the pandemic. When their sense of isolation was exacerbated during the lockdown, “reminding yourself that you actually want to learn this”, “remembering the purpose of why I'm studying”, embracing a “mindset” of “not being fearful of the technology,” and being open to what one can “…learn from this online experience” were persistence manoeuvres that they created to sustain learning engagement. First-year students, who were also adapting to shifting from high school to university, had to convince themselves further to be “… independent … learn and find solutions for some small problems” because “they [the lecturers] can provide the resource, they can provide the skills, they can provide the … support structure, but they cannot do the self-direction … for students.”

Persistence manoeuvres could be facilitated through ‘social support’. Encouragement from family members, having online chats in “virtual hangout space” with friends, and time with room-mates where “we removed the TV… everyone would go to the lounge [and sit on] couches in a circle” were critical during the lockdown. Especially for undergraduates, persistence manoeuvres could be aborted in the absence of social support*.* For example, attending lectures with friends served this function as an undergraduate shared: “… as soon as it [classes] went online [during lockdown] … they [her peers] just didn't have that motivation of going [to online classes]. And so they got behind.”

In students' eyes, persistence manoeuvres motivated them to participate, but they still needed ways of “being there” in spirit, just as they would during face-to-face learning. Therefore, students used different learning presence manoeuvres to sustain their concentration and attention. For on-campus students, using learning presence manoeuvres to manage screen fatigue during lockdowns was critical because they attended live classes or watched lecture recordings throughout the day. A student shared, “I just wasn’t able to concentrate if I [viewed online] lectures one after the other.” Therefore, when this student had back-to-back online lectures, he would “go to the 9 to 12 [online class]. And then I just missed the 12 to 1, and I go back and watch it because I can’t sit there for four hours.” In this respect, lecturers who presented with good ‘showmanship’ helped. This meant that lecturers exuded passion, spoke at the “students’ level and understanding,” made lectures “more entertaining,” “very catchy” and used “good slides that are not too full of information.” Such lecturers gave students “something to bring you back into the [the class session].” Conversely, poor technical control disrupted concentration. For example, “[the lecturers] turn their head away from the microphone, and suddenly it is like 15 minutes of silence.”

Interestingly, distance learning students aimed for more than just focus and concentration through their learning presence manoeuvres. To them, online learning engagement was *just as good* when they had immersive experiences where “I feel like I was in a classroom.” These students have been improving their learning presence manoeuvres even before the pandemic*.* Besides upgrading their computing equipment, using larger-sized monitors and noise-cancelling headsets, they dressed as if they were attending class in person. Efforts were made to improve personal online participation behaviours because they wanted to “be comfortable while also allowing for the comfort of the other people.” Therefore, these students trained themselves to be “confident with speaking in front of others,” avoided being overly dominant in class, paused and allowed for audio transmission lags where “the people [whose] faces you can’t see … wants to speak.” When participation from other students was not forthcoming, some even took the initiative to “start asking questions … to provoke a bit of thought so [that] we can get other students involved.” They knew that “if everyone is not talking, the norm is no conversation at all.” These learning presence manoeuvres contrasted greatly with on-campus undergraduate students who paid less attention to their online behaviours. A student shared, “…people might not initially have shown up [to class] wearing pyjamas, but further on they stopped caring [because] it's 9 a.m. … [I] get myself breakfast, and then just sit there with my camera off until I finish.” During Zoom™ breakout sessions, they remained reticent because “… we didn't know each other. It was really awkward… you'd just be sitting there for 10 minutes, and no one would say a word.”

How lecturers enhanced ‘approachability’ by creating an inviting and conducive learning atmosphere was necessary to encourage students to pay attention to their learning presence manoeuvres. Students described this as “it was more like she [the lecturer] was sitting there having a chat with us.” Approachability could also make lessons “more in-person and engaging” by encouraging enough students to turn on their cameras. Else, even active students became discouraged as “it is quite isolating to feel like you're just talking to an empty room… I am the only person asking questions … it gets quite wanting.” By and by, “[if] nobody would start to talk and everyone had their camera and the microphone off, then I will just follow the group.”

## Discussion

We employed a grounded theory approach, collecting interview data from 32 higher education students to conceptualise online learning dexterity from their pandemic learning experiences.

### Nature of Online Learning Dexterity

Online learning literature tends to focus on the self-regulatory aspects of online learning. This concerns how students sustain pre-determined goals for learning tasks through systematic cycles of forethought, performance, and self-reflection (Neroni et al., 2019; Zimmerman, 2002). The study findings show that some characteristics of self-regulation were reflected in students’ enactment of online learning dexterity for productivity. For example, students could systematically set goals and refine their strategies to become more efficient with institutional technologies. Nevertheless, Pilling-Cormick and Garrison (2007) observe that self-regulation focuses on the internal processes for self-managing study processes but does not consider how the learning environment may influence these processes. We found that contextual factors highly influence online learning dexterity. For example, the usability of institutional technology systems and how lecturers orchestrated accessibility and predictability in their online courses affected how students used time optimisation manoeuvres. Students had to constantly monitor and respond to changes in lecturers’ teaching and assessment plans with new ways of time management.

In contrast to the systematic and cyclical self-regulatory processes visualised by Zimmerman (2002), the enactment of online learning dexterity by the study participants was often episodic. When faced with changing task conditions, students needed adaptive expertise to modify their strategies to sustain good performance (Hatano & Inagaki, 1986). For example, postgraduates re-used their established deep learning manoeuvres for note-taking and revision, modifying some aspects to improve efficacy by considering the problems they encountered during video conferencing. Therefore, online learning dexterity is at times goal-directed doing and at times impromptu action.

### Quality of Online Learning Dexterity

The findings suggest that online learning dexterity helps sustain productivity, efficacy, and engagement during online learning. Students with prior online learning experiences may demonstrate better dexterity because domain knowledge supports adaptiveness (Bohle Carbonell et al., 2014). Most postgraduate study participants were enrolled in their distance learning programmes before the pandemic. In comparison, on-campus undergraduates lacked knowledge of online collaboration, saw learning efficacy as content acquisition, with teachers being the authoritative source, and were forced by circumstances to study online (Dodson & Blinn, 2021). Learning motivation had a weak relationship with online learning engagement (Alemayehu & Chen, 2021), but the pandemic situation may have changed this. Undergraduates’ experiences and motivations for online learning could be why they made minimal adjustments to their learning presence manoeuvres, chose to remain non-participatory during breakout room discussions, and relied on student-instructor interaction for support (Moore, 1989). Like many students during emergency remote learning (e.g. Yeung & Yau, 2021), the undergraduate study participants could generate persistence manoeuvres, adjust time optimisation manoeuvres, continue using familiar study strategies, and switch support-seeking manoeuvres from face-to-face to email exchange with lecturers. These strategies effectively sustained participation and learning outcomes during the pandemic (Koh & Daniel, 2022), but their failure to adjust learning presence manoeuvres and support-seeking manoeuvres resulted in frigid learning atmospheres (Castelli & Sarvary, 2021). This, in turn, contributed to their engagement challenges and made it difficult for them to conquer the communicative challenges of online learning environments (Marshalsey & Sclater, 2020; Symeonides & Childs, 2015).

Online learning competencies are developed over time (Lee et al., 2019), and postgraduates tend to have better self-regulation abilities than undergraduates (Tseng et al., 2019). The postgraduate study participants had more sophisticated deep learning manoeuvres and support-seeking manoeuvres as they focused on a critical understanding of information and used more learning support strategies (Mayer, 1988; McCombs, 2017). They also behaved liked adaptive experts who continually improved their strategies by learning from the different situations they encountered (Mylopoulos et al., 2018). The postgraduates were more contextually aware. While learning management system quality influenced students’ time optimisation manoeuvres (Koh & Kan, 2020), postgraduates exploited discussion boards as online resources to expand their deep learning manoeuvres through student-content interaction (Moore, 1989). Lecturers’ showmanship is a teaching presence factor (Garrison et al., 2010), and how they orchestrate course policies and lesson design could influence students’ enactment of learning manoeuvres. Postgraduates chose to work with contexts by reflecting on their online learning experiences, redefining engagement goals, improving their learning presence manoeuvres with better equipment and online interaction behaviours, and fostering approachability during Zoom™ sessions. In so doing, they contributed to creating cohesive online learning climates to encourage social presence and adaptation (Bohle Carbonell et al., 2014; Garrison et al., 2010). These aspects were significant for sustaining learning engagement during the pandemic (Conklin & Dikkers, 2021).

### Relevance and Implications of Online Learning Dexterity for Higher Education

There are several implications for higher education teaching and learning. before the pandemic, it was suggested that higher education students should enrol in online learning courses after they have developed a baseline level of online readiness (Dray et al., 2011). The study findings show that institutions’ online learning endeavours need not be constrained as such when students have good online learning dexterity to adjust and improve learning manoeuvres according to their online learning situations.

Firstly, as task goals influence how people adapt (Bohle Carbonell et al., 2014), students’ learning goals influence their enactment of online learning dexterity. Therefore, institutions need to set clear visions for the post-pandemic role of online learning, mainly how the affordances of online systems could be exploited to make learning experiences ‘even better.’ Institutions can support students in formulating learning goals through a deeper appreciation of online learning as critical inquiry occurs within online learning communities (Garrison et al., 2010; Gunawardena et al., 1997). Institutions can also help students benefit from learning analytics, content creation tools and social networking tools to learn in personalised and community-supported ways (Koh & Kan, 2021). These conceptions of online learning can help students to visualise new ways of enacting learning manoeuvres that are not yet evident in their practices. Clarifying the institutional vision for online learning also facilitates the identification of the online learning dexterities that may be critical for student success in the institutional context.

Secondly, the study results show that competencies for online learning cannot be divorced from their contexts of use (Alvarez et al., 2009). Therefore, institutions need to develop learning environments that facilitate online learning dexterity. Technically, this could mean selecting learning technologies that enhance students’ online learning efficiency and integrating learning management systems with interoperable tools to facilitate quick resource access and deep learning (Koh & Kan, 2021). It could also involve ways of enhancing lecturers’ ability to orchestrate online learning well. Good showmanship and capabilities for designing online courses with approachability, predictability, and accessibility characterise the practices of exemplary online teachers (Baran et al., 2013). Besides using workshop programmes to develop an understanding of these practices, institutions also need to keep abreast of emerging online teaching competencies and provide instructional consultation to support lecturers’ actualisation of effective practices (Koh, 2020).

Thirdly, institutions need to better understand how students enact online learning dexterity by analysing their learning manoeuvres, the contextual facilitators, and the barriers they encounter. The findings show that students’ enactment of learning manoeuvres is closely related to their competencies in areas such as time management, note-taking, and self-regulation. Institutions must develop study competencies and students’ adaptive expertise for using competencies with dexterity during online learning (Alvarez et al., 2009; Hatano & Inagaki, 1986). Just as professionals learn to adapt by exploring solutions in different situations (Mylopoulos et al., 2018), academic development programmes need to enhance students’ ability to solve different kinds of online learning problems through encountering authentic situations, creating learning manoeuvres, testing, and reflecting upon their problem-solving strategies.

## Limitations and Future Research

This study has several limitations that may also serve as areas for future research. Firstly, this study focused on the pandemic learning experiences of students in a New Zealand university. The university programme policies and the New Zealand pandemic health advisories could have influenced how students and lecturers approached online learning and teaching. While students’ experiences during the study period provided insights about online learning both during the lockdown and when students returned to campus after lockdown, the framework of online learning dexterity presented in this study still needs to be further validated in other institutions' programme contexts and during post-pandemic conditions. Since grounded theory is based upon theoretical sampling and study participation was voluntary, not all possible demographic profiles were covered in the present study. Therefore, the transferability of the findings needs to be validated with participants outside of the sampled demographic profiles. Secondly, this study is an initial attempt to demarcate what online learning dexterity could mean from students’ learning experiences. Further analyses of its theoretical linkages with self-regulation and adaptive expertise are needed. More in-depth qualitative studies and survey designs are also needed to validate the students’ online learning manoeuvres and contextual facilitators and barriers outlined in this study. Thirdly, survey studies have found that students with higher self-efficacy, conscientiousness, and openness to new experiences have better online learning performance (Bahçekapili & Karaman, 2020). The study focus did not allow us to examine these aspects. In future studies, online readiness surveys could be used to identify students’ dispositions and their possible effects on online learning dexterity. Fourthly, the learning environment factors in this study were based on students’ perspectives of lecturer strategies. In future studies, lecturers’ and students’ online dexterity could be examined within a course to pinpoint how lecturers’ orchestration of learning environments influenced students’ online learning manoeuvres more accurately. Finally, the study focus did not allow us to track the development of students’ online learning dexterity. Longitudinal studies could be conducted to better understand how students developed online learning dexterity throughout their university studies. Through such initiatives, academic development programmes can be better customised towards the online learning dexterity of different student groups.

## Conclusions

While online learning dexterity is a critical construct that enables us to understand students’ competency for learning effectively and independently online, its relevance, value, and scope depend on institutional contexts. Students’ online dexterity is driven by understanding the intricacies of engagement and support, but most importantly, how the dexterities of students, teachers, and the institution interact. These different aspects of online dexterity critically affect how higher education institutions create higher quality teaching and learning beyond the pandemic. A more comprehensive view of these different forms of dexterity needs to be considered.

## Data Availability

The data is not publicly available. Please contact the corresponding author.

## References

[CR1] Alemayehu, L., & Chen, H.-L. (2021). The influence of motivation on learning engagement: The mediating role of learning self-efficacy and self-monitoring in online learning environments. *Interactive Learning Environments*. 10.1080/10494820.2021.1977962

[CR2] Alvarez, I., Guasch, T., & Espasa, A. (2009). University teacher roles and competencies in online learning environments: A theoretical analysis of teaching and learning practices. *European Journal of Teacher Education,**32*(3), 321–336. 10.1080/02619760802624104

[CR4] Bahçekapili, E., & Karaman, S. (2020). A path analysis of five-factor personality traits, self-efficacy, academic locus of control and academic achievement among online students. *Knowledge Management & E-Learning,**12*(2), 191–208.

[CR5] Baran, E., Correia, A.-P., & Thompson, A. (2013). Tracing successful online teaching in higher education: Voices of exemplary online teachers. *Teachers College Record, 115*(3), 1–41. https://www.tcrecord.org/Content.asp?ContentId=16896

[CR6] Biwer, F., Wiradhany, W., Oude Egbrink, M., Hospers, H., Wasenitz, S., Jansen, W., & de Bruin, A. (2021). Changes and adaptations: How university students self-regulate their online learning during the COVID-19 pandemic. *Frontiers in Psychology,**12*, 642593–642593. 10.3389/fpsyg.2021.642593 33967903 10.3389/fpsyg.2021.642593 PMC8103204

[CR7] Bohle Carbonell, K., Stalmeijer, R. E., Könings, K. D., Segers, M., & van Merriënboer, J. J. G. (2014). How experts deal with novel situations: A review of adaptive expertise. *Educational Research Review,**12*, 14–29. 10.1016/j.edurev.2014.03.001

[CR8] Broadbent, J., & Poon, W. L. (2015). Self-regulated learning strategies & academic achievement in online higher education learning environments: A systematic review. *The Internet and Higher Education,**27*, 1–13. 10.1016/j.iheduc.2015.04.007

[CR9] Castelli, F. R., & Sarvary, M. A. (2021). Why students do not turn on their video cameras during online classes and an equitable and inclusive plan to encourage them to do so. *Ecology and Evolution,**11*(8), 3565–3576. 10.1002/ece3.7123 33898009 10.1002/ece3.7123 PMC8057329

[CR10] Chan, B. C., Baker, J. L., Bunagan, M. R., Ekanger, L. A., Gazley, J. L., Hunter, R. A., O’Connor, A. R., & Triano, R. M. (2020). Theory of Change to Practice: How experimentalist teaching enabled faculty to navigate the COVID-19 disruption. *Journal of Chemical Education,**97*(9), 2788–2792. 10.1021/acs.jchemed.0c00731

[CR11] Charmaz, K. (2006). *Constructing grounded theory: A practical guide through qualitative analysis*. SAGE. http://ebookcentral.proquest.com/lib/otago/detail.action?docID=585415

[CR12] Conklin, S., & Dikkers, A. G. (2021). Instructor social presence and connectedness in a quick shift from face-to-face to online instruction. *Online Learning,**25*(1), 135–150. 10.24059/olj.v25i1.2482

[CR13] Corbin, J. M., & Strauss, A. (1990). Grounded theory research: Procedures, canons, and evaluative criteria. *Qualitative Sociology,**13*(1), 3–21. 10.1007/BF00988593

[CR14] Creswell, J. W. (1998). *Qualitative inquiry and research design*. SAGE.

[CR15] Dietrich, N., Kentheswaran, K., Ahmadi, A., Teychene, J., Bessiere, Y., Alfenore, S., Laborie, S., Bastoul, D., Loubiere, K., Guigui, C., Sperandio, M., Barna, L., Paul, E., Cabassud, C., Line, A., & Hebrard, G. (2020). Attempts, successes, and failures of distance learning in the time of COVID-19. *Journal of Chemical Education,**97*(9), 2448–2457. 10.1021/acs.jchemed.0c00717

[CR16] Dodson, E. M., & Blinn, C. R. (2021). Forest operations instructor and student perspectives on rapid transition from face-to-face to online learning in the US. *International Journal of Forest Engineering*. 10.1080/14942119.2021.1907109

[CR17] EDUCAUSE. (2022). EDUCAUSE Horizon Report—Teaching and Learning Edition. https://library.educause.edu/-/media/files/library/2022/4/2022hrteachinglearning.pdf?la=en&hash=6F6B51DFF485A06DF6BDA8F88A0894EF9938D50B

[CR18] Garrison, D. R., Anderson, T., & Archer, W. (2010). The first decade of the community of inquiry framework: A retrospective. *The Internet and Higher Education,**13*(1), 5–9. 10.1016/j.iheduc.2009.10.003

[CR19] Gerhart, L. M., Jadallah, C. C., Angulo, S. S., & Ira, G. C. (2021). Teaching an experiential field course via online participatory science projects: A COVID-19 case study of a UC California Naturalist course. *Ecology and Evolution,**11*(8), 3537–3550. 10.1002/ece3.7187 33821180 10.1002/ece3.7187 PMC8013971

[CR20] Gunawardena, C. N., Lowe, C. A., & Anderson, T. (1997). Analysis of a global online debate and the development of an interaction analysis model for examining social construction of knowledge in computer conferencing. *Journal of Educational Computing Research,**17*(4), 397–431. 10.2190/7mqv-x9uj-c7q3-nrag

[CR21] Hatano, G., & Inagaki, K. (1986). Two courses of expertise. In H. A. H. Stevenson & K. Hakuta (Eds.), *Child development and education in Japan* (pp. 262–272). Freeman.

[CR22] Hu, H., & Gramling, J. (2009). Learning strategies for success in a web-based course: A descriptive exploration. *Quarterly Review of Distance Education, 10*(2), 123–134, 250. https://www.proquest.com/scholarly-journals/learning-strategies-success-web-based-course/docview/231183082/se-2?accountid=14700

[CR23] Koh, J. H. L. (2020). Three approaches for supporting faculty technological pedagogical content knowledge (TPACK) creation through instructional consultation. *British Journal of Educational Technology,**51*(6), 2529–2543. 10.1111/bjet.12930

[CR24] Koh, J. H. L., & Kan, R. Y. P. (2020). Perceptions of learning management system quality, satisfaction, and usage: Differences among students of the arts. *Australasian Journal of Educational Technology,**36*(3), 26–40. 10.14742/ajet.5187

[CR25] Koh, J. H. L., & Kan, R. Y. P. (2021). Students’ use of learning management systems and desired e-learning experiences: Are they ready for next generation digital learning environments? *Higher Education Research & Development,**40*(5), 995–1010. 10.1080/07294360.2020.1799949

[CR43] Koh, J. H. L., & Daniel, B. K. (2022). Shifting online during COVID-19: A systematic review of teaching and learning strategies and their outcomes. *International Journal of Educational Technology in Higher Education, 19*(1), 56. 10.1186/s41239-022-00361-7 10.1186/s41239-022-00361-7 PMC9643977 36404984

[CR26] Lee, K., Choi, H., & Cho, Y. H. (2019). Becoming a competent self: A developmental process of adult distance learning. *The Internet and Higher Education,**41*, 25–33. 10.1016/j.iheduc.2018.12.001

[CR27] Marshalsey, L., & Sclater, M. (2020). Together but apart: Creating and supporting online learning communities in an era of distributed studio education. *International Journal of Art & Design Education,**39*(4), 826–840. 10.1111/jade.12331

[CR28] Mayer, R. E. (1988). 2 - LEARNING STRATEGIES: AN OVERVIEW. In C. E. Weinstein, E. T. Goetz, & P. A. Alexander (Eds.), *Learning and study strategies* (pp. 11–22). Academic Press. 10.1016/B978-0-12-742460-6.50008-6

[CR29] McCombs, B. L. (2017). Historical Review Of Learning Strategies Research: Strategies For The Whole Learner—a tribute to Claire Ellen Weinstein and early researchers of this topic. *Frontiers in Education*. 10.3389/feduc.2017.00006

[CR30] Merriam-Webster. (n.d.). *Dexterity*. Retrieved December 8, 2021, from https://www.merriam-webster.com/dictionary/dexterity

[CR31] Moore, M. G. (1989). Three types of interaction. *American Journal of Distance Education,**3*(2), 1–7. 10.1080/08923648909526659

[CR32] Mylopoulos, M., Kulasegaram, K., & Woods, N. N. (2018). Developing the experts we need: Fostering adaptive expertise through education. *Journal of Evaluation in Clinical Practice,**24*(3), 674–677. 10.1111/jep.12905 29516651 10.1111/jep.12905

[CR33] Neroni, J., Meijs, C., Gijselaers, H. J. M., Kirschner, P. A., & de Groot, R. H. M. (2019). Learning strategies and academic performance in distance education. *Learning and Individual Differences,**73*, 1–7. 10.1016/j.lindif.2019.04.007

[CR34] Pilling-Cormick, J., & Garrison, D. R. (2007). Self-directed and self-regulated learning: Conceptual links. *Canadian Journal of University Continuing Education,**33*(2), 13–33. 10.21225/D5S01M

[CR35] Sebastian, K. (2019). Distinguishing between the strains grounded theory: Classical, interpretive and constructivist. *Journal for Social Thought, 3*(1). https://ojs.lib.uwo.ca/index.php/jst/article/view/4116

[CR36] Singh, V., & Thurman, A. (2019). How many ways can we define online learning? A systematic literature review of definitions of online learning (1988–2018). *American Journal of Distance Education,**33*(4), 289–306. 10.1080/08923647.2019.1663082

[CR37] Symeonides, R., & Childs, C. (2015). The personal experience of online learning: An interpretative phenomenological analysis. *Computers in Human Behavior,**51*, 539–545. 10.1016/j.chb.2015.05.015

[CR38] Tseng, H., Yi, X., & Yeh, H.-T. (2019). Learning-related soft skills among online business students in higher education: Grade level and managerial role differences in self-regulation, motivation, and social skill. *Computers in Human Behavior,**95*, 179–186. 10.1016/j.chb.2018.11.035

[CR39] Unite against COVID-19. (2022). *History of the COVID-19 Alert System*. https://covid19.govt.nz/about-our-covid-19-response/history-of-the-covid-19-alert-system/

[CR40] Yeung, M. W. L., & Yau, A. H. Y. (2021). A thematic analysis of higher education students’ perceptions of online learning in Hong Kong under COVID-19: Challenges, strategies and support. *Education and Information Technologies*. 10.1007/s10639-021-10656-3 10.1007/s10639-021-10656-3 PMC8364774 34421326

[CR41] Yundayani, A., Abdullah, F., Tandiana, S. T., & Sutrisno, B. (2021). Students’ cognitive engagement during emergency remote teaching: Evidence from the Indonesian EFL milieu. *Journal of Language and Linguistic Studies,**17*(1), 17–33. 10.52462/jlls.2

[CR42] Zimmerman, B. J. (2002). Becoming a self-regulated learner: An overview. *Theory into Practice, 41*(2), 64. https://www.proquest.com/scholarly-journals/becoming-self-regulated-learner-overview/docview/218832636/se-2?accountid=14700

